# Granulomatosis with polyangiitis presenting as a solitary renal mass: a case report

**DOI:** 10.1186/s12882-023-03276-1

**Published:** 2023-07-28

**Authors:** Mai Higashihara, Tetsuya Kawamura, Yoichi Miyazaki, Takashi Yokoo, Kensuke Joh

**Affiliations:** 1 Division of Nephrology and Hypertension, Department of Internal Medicine, The Jikei University School of Medicine, 3-25-8 Nishi-Shinbashi, Minato-Ku, Tokyo, Japan; 2 Department of Pathology, The Jikei University School of Medicine, Tokyo, Japan

**Keywords:** Granulomatosis with polyangiitis, Inflammatory pseudotumor, Kidney, Solitary mass, Vasculitis, Case report

## Abstract

**Background:**

Granulomatosis with polyangiitis (GPA) is characterized by necrotizing granulomatous vasculitis involving small-sized vessels in the upper airways, lower airways, and kidneys. Renal pathology is usually characterized by focal segmental necrotizing glomerulonephritis, which often leads to rapidly progressive renal failure. This type of renal involvement is usually unapparent on radiography. The presence of a renal mass in a patient with GPA, although extremely rare, is recognizable. Herein, we report a rare case of GPA presenting as a solitary renal mass and present a review of the literature.

**Case presentation:**

A 75-year-old woman presented with a solitary right kidney mass measuring 4 × 3.5 cm detected by enhanced computed tomography. There was no history of sinusitis, rhinitis, cough, or pneumonia suggestive of systemic GPA. Nephrectomy was performed based on the suspicion of renal cell carcinoma or malignant lymphoma. Three months later, she was admitted because her serum creatinine levels increased from 54.81 μmol/L to 405.76 μmol/L accompanied by a high C-reactive protein level of 159 mg/L. Anti-neutrophil cytoplasmic antibodies against myeloperoxidase and anti-proteinase 3 were negative. Histological examinations of the solitary renal mass revealed necrotizing granulomatous arteritis in the cortex and medullary vasa recta, surrounded by interstitial fibrosis, and focal segmental necrotizing glomerulonephritis in the localized lesion; however, signs of vasculitis were not observed in areas other than the solitary mass. Therefore, the patient was diagnosed with granulomatosis with polyangiitis (GPA). Despite treatment with prednisolone (30 mg/day), the patient developed oliguria with an elevation of her serum creatinine level to 583.44 μmol/L, which required hemodialysis within one month after the initiation of steroid therapy. The patient could successfully discontinue hemodialysis 21 months later, following a decrease in her serum creatinine level to 251.06 μmol/L.

**Conclusions:**

GPA should be considered as one of the differential diagnoses of a solitary renal mass. Furthermore, patients with solitary renal masses associated with GPA may exhibit a favorable response to steroid or immunosuppressive treatment.

## Background

Granulomatosis with polyangiitis (GPA) is a systemic disease characterized by necrotizing granulomatous vasculitis involving small-sized vessels in the upper airways, lower airways, and kidneys [1]. The characteristic pathological features of GPA are focal segmental necrotizing glomerulonephritis with crescents and granulomatous arteritis affecting the entire kidney, according to the 2012 Revised International Chapel Hill Consensus Conference (CHCC) Nomenclature of Vasculitides [2]. The renal involvement observed in GPA is not usually visible with imaging techniques [3], although tumorous masses commonly manifest in the upper respiratory tracts or lungs of patients with GPA [4]. However, the presence of a renal mass as a presentation of GPA has rarely been reported [5]. Furthermore, renal masses in GPA can be solitary or multiple and unilateral, or bilateral [6, 7]. Herein, we report a case of GPA presenting as a solitary renal mass resembling a renal tumor and discuss this rare presentation with a review of the literature.

## Case presentation

**Fig. 1:**
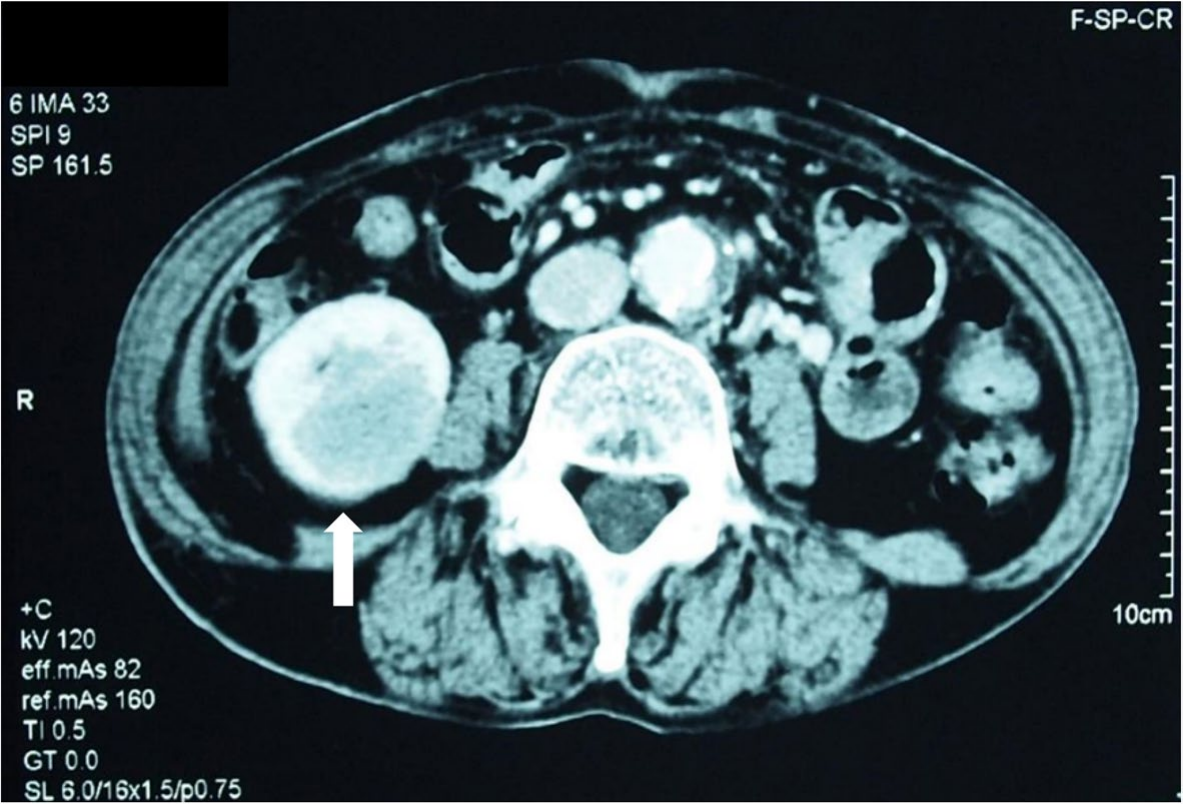
Contrast-enhanced computed tomographic scan showing a low-density right renal mass (arrow). In the inferior pole of the right kidney, a solitary and localized area of interstitial fibrosis measuring 40 × 35 × 30 mm is observed to extend from the cortex to the medulla

A 75-year-old female patient was found to exhibit microhematuria during a medical checkup and referred to the outpatient urology clinic of our hospital. Her serum creatinine and C-reactive protein (CRP) levels were 54.81 μmol/L and 70 mg/L, respectively. Urinary sediment examinations revealed granular casts and 5–9 red blood cells/high-power field. There was no history of sinusitis, rhinitis, cough, or pneumonia suggestive of systemic GPA. There was no history of other autoimmune diseases, chronic renal failure, bronchiectasis, pneumonia, sinus infection, or smoking. Brain magnetic resonance imaging and chest computed tomography did not reveal any findings in the upper respiratory tract or lungs that were suggestive of GPA. Contrast-enhanced computed tomography revealed a solitary mass measuring 4 × 3.5 cm in the inferior pole of the right kidney (Fig. 1). Renal cell carcinoma or malignant lymphoma was suspected, and a right nephrectomy was performed.

Three months after surgery, the patient developed a loss of appetite and was transferred from the urology unit to the nephrology unit and readmitted for further examination. On admission, there were no abnormalities in the upper and lower respiratory tracts. Laboratory data revealed elevated serum creatinine and CRP levels (405.76 μmol/L and 159 mg/L, respectively) and white blood cell counts (16.5 × 10^9^/L) with reduced hemoglobin levels (72 g/L) (Table 1). The patient tested negative for anti-neutrophil cytoplasmic antibodies (ANCA) against myeloperoxidase (MPO) and anti-proteinase 3 (PR3). Her urinary protein excretion was 0.23 g/day, and urinary sediment examinations revealed granular casts and 10–19 red blood cells/high-power field.

**Table 1:** Laboratory findings on admission

**(Complete blood count)**		Fe	2.15 μmol/L	ANA (EIA)	28.8
WBC	16.5 × 10^9^/L	Ferritin	277 μg/L	Anti-dsDNA-IgG	< 5 IU/mL
Neutrophils	14.9 × 10^9^/L	**(Infection markers)**		Anti-Sm Ab	−
Lymphocytes	0.97 × 10^9^/L	HBs Ag	0.01 IU/mL	Anti-SS-A Ab	−
Monocytes	0.5 × 10^9^/L	HCV Ab	0.16 S/CO	Anti-SS-B Ab	−
Eosinophils	0.1 × 10^9^/L	**(Immunological markers)**		**(Urine biochemistry)**	
Basophils	0.03 × 10^9^/L	IgG	21.16 g/L	24 h Ccr	0.08 mL/s/m^2^
Red blood cell count	2.54 × 10^12^/L	IgG4	0.91 g/L	Urinary protein level	0.23 g/day
Hemoglobin	72 g/L	IgA	6.95 g/L	(Urinalysis)	
Platelet count	606 × 10^9^/L	IgM	0.61 g/L	Protein	+ 1
**(Biochemistry)**		C3	1.22 g/L	Sugar	−
TP	72 g/L	C4	0.26 g/L	Occult blood	±
Alb	21 g/L	CH50	56.1 U/mL	**(Urinary sediment)**	
UN	18.56 mmol/L	MPO-ANCA	< 10 EU	Red blood count	10–19/HPF
Cr	405.76 μmol/L	PR3-ANCA	< 10 EU	White blood count	5–9/HPF
CRP	159 mg/L			Granular cast	+ 1
Procalcitonin	0.60 ng/mL			Dysmorphic red blood cells	−

*Ab* antibody, *ANA* antinuclear antibody, *ANCA* anti-neutrophil cytoplasmic antibody, *Alb* albumin, *Ccr* creatinine clearance, *CH50* 50% hemolytic complement activity, *Cr* creatinine, *CRP* C-reactive protein, *EIA* enzyme immunoassay, *Fe* ferrum, *HBs Ag* hepatitis B surface antigen, *HCV Ab* hepatitis C virus antibody, *HPF* high-power field, *MPO* myeloperoxidase, *PR3* proteinase 3, *Sm* Smith, *SS-A* Sjögren's syndrome-A, *SS-B* Sjögren's syndrome-B, *TP* total protein, *UN* urea nitrogen

**Fig. 2:**
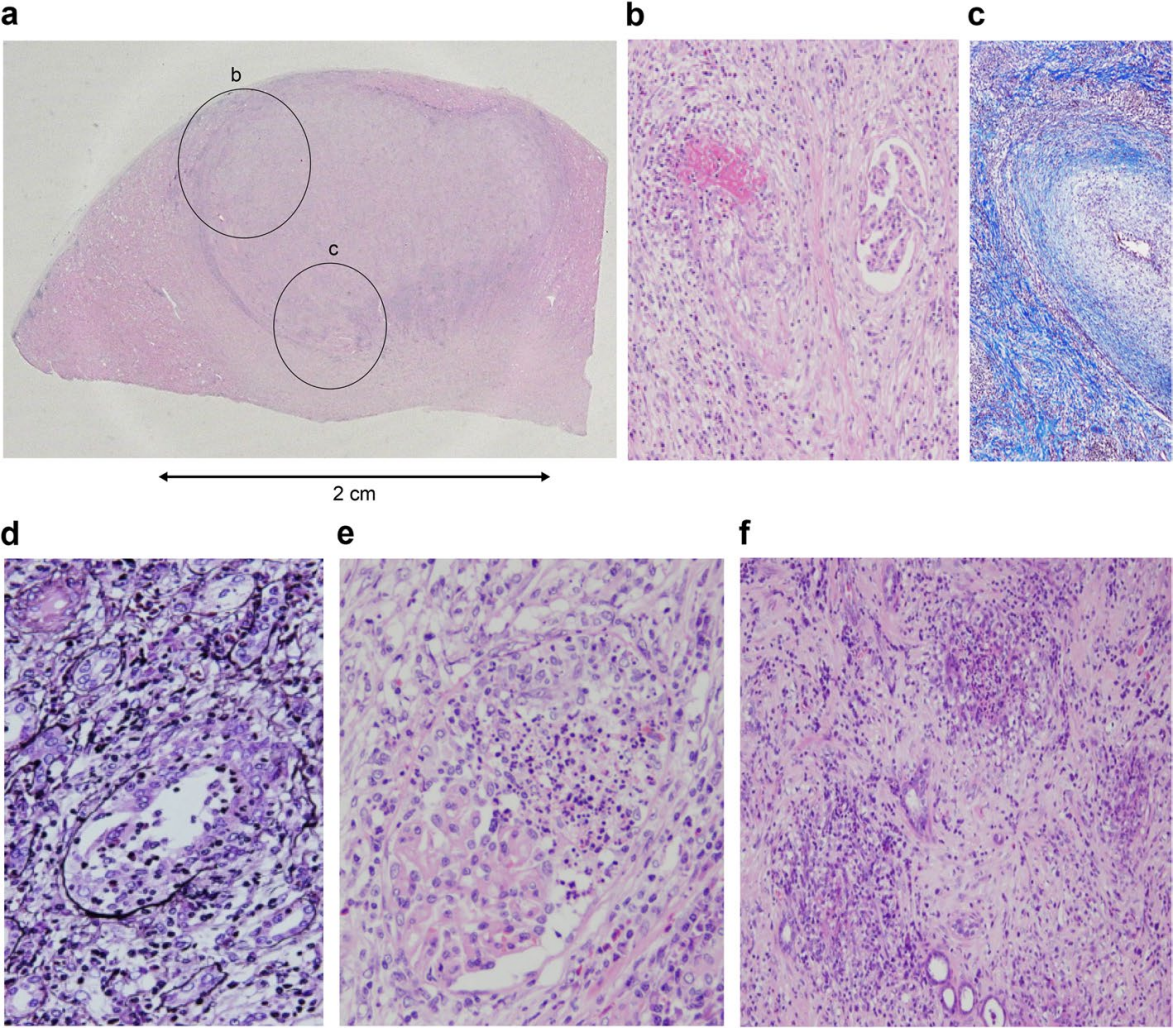
**a** A solitary and localized fibrotic interstitium measuring 40 × 35 × 30 mm extending from the cortex to the medulla. Note lymphocytic infiltration with lymph follicle formation at the demarcated boundary of the lesion (circle b). A higher magnification of the area indicated by the small circle, c, is shown in Fig. 2b. (hematoxylin/eosin-stain × 1). **b** Fibrinoid necrosis of interlobular artery, which is situated in the periphery of the extensively fibrotic area. Macrophagic infiltration is observed around the area with fibrinoid necrosis, whereas endarteritis is also seen inside the necrotic medial layer toward the arterial lumen (hematoxylin/eosin-stain × 100). **c** The arcuate artery supplying the blood flow to the fibrotic area shows severe intimal fibrosis, ruling out elastofibrosis due to hypertension, resulting in remarkable narrowing of the vascular lumen (Masson trichrome stain × 100).** d** Granulomas were found in the damaged area of the destructive lesion on the tubular basement membrane, which was located further away from granulomatous arteritis (periodic acid–methenamine–silver stain × 200). **e** Focal segmental necrotizing glomerulonephritis with neutrophilic infiltration (hematoxylin/eosin-stain × 200). **f** Necrotizing vasculitis with neutrophilic infiltration is observed primarily around the medullary vasa recta (Periodic acid–Schiff stain × 100)

Because of the suspicion of the presence of other non-tumoral diseases, further histological analyses of the excised kidney were performed by the renal pathologist, who identified a solitary and localized fibrotic mass measuring 40 × 35 × 30 mm, extending from the cortex to the medulla (Fig. 2a). Inside the lesion, necrotizing arteritis with fibrinoid necrosis was surrounded by macrophagic infiltration and interstitial fibrosis with multiple lymph follicles. Endarteritis was observed within the necrotic medial layer toward the arterial lumen. These findings were consistent with granulomatous arteritis (Fig. 2b). The arcuate artery supplying blood to the fibrotic area showed severe intimal fibrosis with remarkable narrowing of the vascular lumen. This finding was consistent with neointima formation and not elastofibrosis due to hypertension (Fig. 2c). Granulomas were also found in the damaged area of the destructive lesion on the tubular basement membrane, which was located away from the areas exhibiting signs of granulomatous arteritis (Fig. 2d). Glomeruli in the localized fibrotic area showed focal segmental necrotizing glomerulonephritis (Fig. 2e). Necrotizing vasculitis with neutrophilic infiltration was also observed primarily around the medullary vasa recta (Fig. 2f). These pathological findings were consistent with the criteria for GPA according to the 2012 International CHCC guidelines [2]. Arteritis and necrotizing glomerulonephritis were not found at sites other than the localized fibrotic area.

**Fig. 3:**
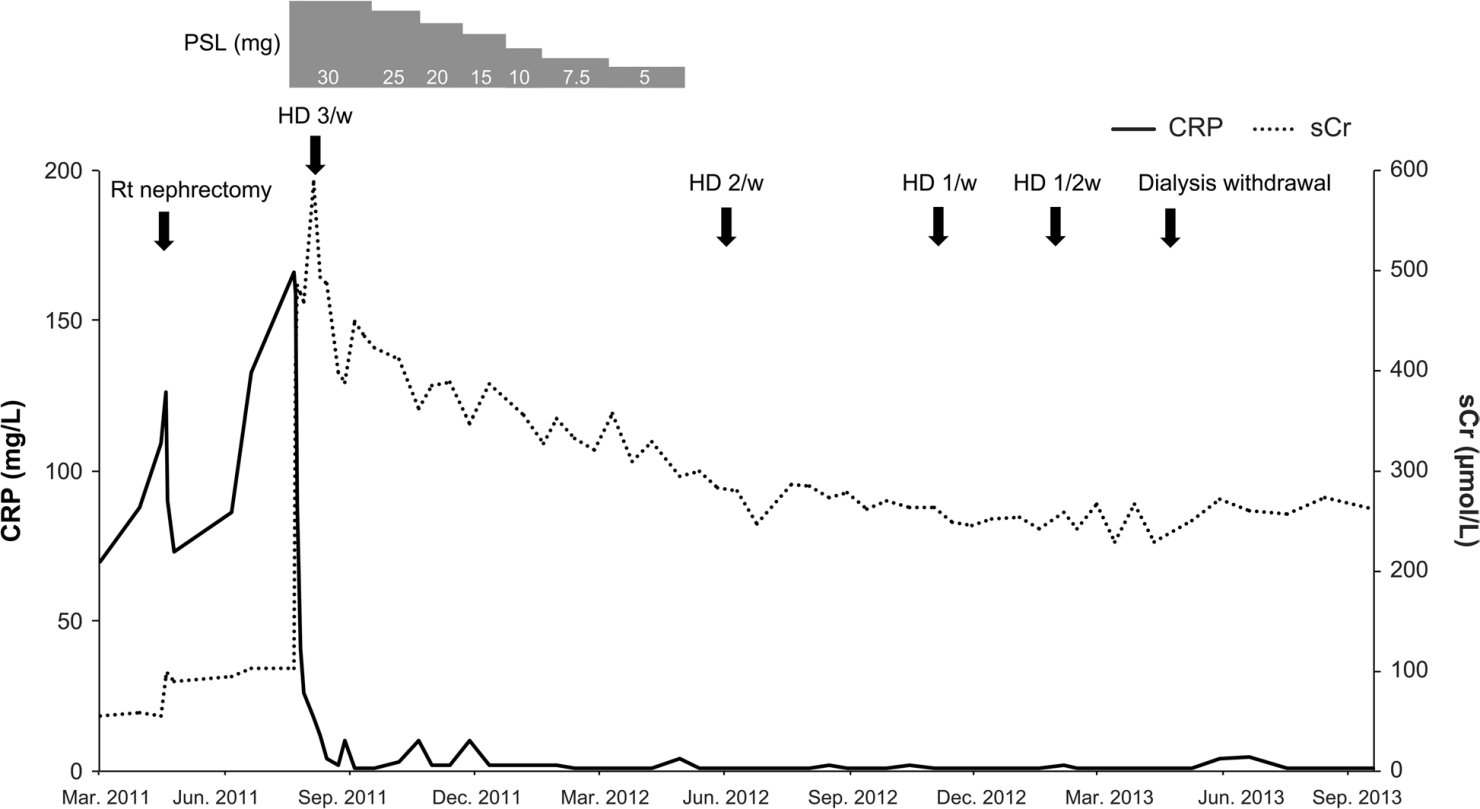
Clinical course of the patient. CRP: C-reactive protein; HD: hemodialysis; PSL: prednisolone; Rt: right; sCr: serum creatinine

Although treatment with prednisolone (30 mg/day) was initiated after nephrectomy, the patient developed oliguria with elevated serum creatinine (583.44 μmol /L) and required hemodialysis within one month after treatment initiation. No biopsy of her left kidney was performed. Prednisolone was tapered off and discontinued ten months after the induction of hemodialysis. The patient’s serum creatinine level gradually decreased to 251.06 μmol /L, and hemodialysis was stopped eleven months after prednisolone discontinuation (Fig. 3). She has not been on dialysis since then.

## Discussion and conclusions

In the present case, the histopathological examination of the solitary renal mass in the resected right kidney revealed granulomatous necrotizing arteritis surrounded by interstitial fibrosis with multiple lymph follicles, focal segmental necrotizing glomerulonephritis, granulomas at the destructive lesion of the tubular basement membrane, and vasculitis around the medullary vasa recta. These lesions corresponded to the characteristic lesions of GPA per the 2012 International CHCC criteria (according to which GPA is defined as the presence of granuloma) while microscopic polyangiitis (MPA) is defined as the absence of granuloma [2]. The diagnosis of GPA was confirmed using the stepwise algorithm of the 2006 European Medicines Agency Consensus Classification of Vasculitis [8]. However, the present case did not meet the strict criteria for GPA according to the 2022 American College of Rheumatology/European Alliance of Associations for Rheumatology classification, according to which a total score of ≥ 5 was needed for the classification of GPA [9]. IgG4-related kidney disease, another well-known condition that can present as localized inflammatory tumor-like lesions in the kidney, was ruled out because of the presence of vasculitis found in the present case. Moreover, plasmacytic infiltration, primarily comprising IgG1 but not IgG4, was prominent in areas of interstitial fibrosis (data not shown) [4]. The destruction in the kidney was centered on glomeruli, tubuli, and arteries. It also extended to the contiguous zones, resulting in massive tissue destruction. The arcuate artery in the mass, which might have been providing support for the localized fibrotic area, exhibited neointimal thickening of the endothelial cells, which resulted in severe narrowing of the arterial lumen. These findings suggest that the destructive process in granulomatous necrotizing arteritis might have been locally augmented in that area because of ischemic endothelial injury resulting from the narrowing of the corresponding arcuate artery. The exact etiology of severe neointimal thickening of the corresponding arcuate artery was unknown; however, it might be related to the etiology of necrotizing vasculitis. The area outside the renal mass did not exhibit signs of vasculitis or intimal thickening of the arcuate arteries, indicating that endothelial injury and vasculitis in small-sized vessels did not reach the level of histological manifestation in the absence of ischemic endothelial injury, which was locally observed in the mass lesion. In the present case, serum creatinine levels increased further despite the right nephrectomy, suggesting the presence of vasculitis in the remaining left kidney. However, the patient tested negative for both MPO-ANCA and PR3-ANCA. One study reported that 54.6% and 45.5% of Japanese patients with GPA tested positive for MPO-ANCA and PR3-ANCA, respectively, and that only 9.1% of patients were ANCA-negative [10]. Moreover, ANCA sensitivity is particularly lower among patients with active localized lesions, as seen in the present case [1, 11]. Therefore, double-negative ANCA vasculitis was suspected in the present case.

GPA is a multiorgan systemic disease characterized by small-vessel vasculitis with a predilection for the respiratory tract, lungs, and kidneys [12]. Tumorous lesions in GPA have been reported in several organs, including breasts, eyes, mediastinum, central nervous system, ovaries, kidneys, genitourinary tract, and lungs [13]. Kariv et al. reported 79 cases of systemic vasculitis presenting with tumor-like lesions, including 28 (35%) patients with GPA, 17 (22%) with Giant cell arthritis, 12 (15%) with Polyarteritis Nodosa, 12 (15%) with Behçet's disease, and 10 (13%) with various vasculitides [13]. Tumor-like lesions were most commonly found in the breasts (seven patients), followed by kidneys (five patients) [13].

The characteristics of 27 reported cases of GPA with renal masses, including 11 female and 16 male patients, are summarized in Table 2 [1, 3–7, 11, 14–32].

**Table 2:** Summary of the published cases of granulomatosis with polyangiitis with a renal mass

Author	Patient (Age/sex)	ENT involvement	Pulmonary involvement	ANCA	Renal masses	Renal histology			Nephrectomy	Treatment	Renal prognosis
						GN	Granulomas	Vasculitis			
**Leung ** [1]	66 y/m	+	−	MPO	Bilateral	−	+	−	No	PSL; MTX	Normal renal function
**Mohhamadi ** [3]	22 y/f	+	−	NM	Right	NM	+	+	No	Death before treatment initiation	
**Ward ** [4]	48 y/f	+	+	PR3	Right	+	+	−	Total	PSL; CPA; rituximab	NM
**Fairbanks ** [5]	68 y/m	+	+	MPO	Left	−	+	+	No	PSL; MTX	Normal renal function
**Yamamoto ** [6]	60 y/m	−	−	MPO	Left	−	+	+	Total	PSL	Normal renal function
**Tiwari ** [7]	60 y/f	−	−	MPO	Right	+	+	NM	No	PSL; MTX	Normal renal function
**Krambeck ** [11]	61 y/m	+	−	−	Right	−	+	−	Partial	PSL; AZA	Normal renal function
**Forte ** [14]	45 y/m	+	+	NM	Right	+	+	+	Total	PSL; CPA; dialysis	ESRD
**Boubenider ** [15]	45 y/f	−	−	PR3	Right	+	+	+	Partial	Dialysis	ESRD
**Smith ** [16]	52 y/f	+	−	NM	Left	−	+	−	Total	PSL; AZA	NM
**Schydlowsky ** [17]	47 y/m	−	+	NM	Left	+	+	+	Total	PSL; CPA	Normal renal function
**Negi ** [18]	40 y/m	−	+	PR3	Bilateral	NM	NM	NM	No	NM	Renal failure
**Schapira ** [19]	45 y/m	+	+	NM	Left	+	+	−	Partial	PSL; CPA	Proteinuria
**Maguire ** [20]	27 y/f	+	+	PR3	Right	+	+	−	Partial	CPA	NM
**Carazo ** [21]	29 y/m	−	−	PR3	Bilateral	+	+	+	Total	PSL; CPA	Normal renal function
**Verswijvel ** [22]	24 y/m	+	−	PR3	Left	+	NM	NM	No	PSL; CPA	Hematuria
**Frigui ** [23]	59 y/f	+	−	PR3	Bilateral	−	+	+	No	PSL; CPA; MTX	Proteinuria
**Kapoor** [24]	22 y/m	−	−	PR3	Bilateral	+	+	+	No	Dialysis	ESRD
**Vandergheynst** [25]	32 y/m	+	−	PR3	Left	+	+	−	Partial	PSL; CPA	Proteinuria
**Roussou** [26]	72 y/f	+	−	MPO	Left	+	+	−	Total	PSL; CPA	Normal renal function
**D’Hauwe** [27]	14 y/f	+	−	−	Right	−	+	NM	No	PSL; MTX; rituximab	Normal renal function
**Ahmed** [28]	28 y/f	−	+	PR3	Bilateral	+	+	NM	No	PSL; CPA	Normal renal function
**Dufour** [29]	70 y/m	+	+	MPO	Right	+	+	+	Total	PSL; CPA	Renal failure
**Dufour** [29]	67 y/m	−	+	PR3	Left	NM	NM	NM	No	PSL; CPA	Normal renal function
**Lo Gullo** [30]	38 y/m	+	+	PR3	Left	−	+	−	No	PSL; rituximab	NM
**Vandergheynst** [31]	23 y/f	+	−	MPO	Bilateral	+	+	−	No	PSL; rituximab	Normal renal function
**Hong** [32]	55 y/m	−	−	PR3	Left	+	NM	NM	Total	PSL	NM
**Present case**	75 y/f	−	−	−	Right	+	+	+	Total	PSL; dialysis	Renal failure

*AZA* azathioprine, *CPA* cyclophosphamide, *ENT* ear, nose, and throat, *ESRD* end-stage renal disease, *f* female, *GN* glomerulonephritis, *m* male, *MPO* anti-myeloperoxidase, *MTX* methotrexate, *NM* not mentioned, *PR3* anti-proteinase 3, *PSL* prednisolone, *y* years

All cases were diagnosed as GPA based on the clinical features at presentation that fulfilled the CHCC criteria [2]. The median age was 45 years, which was lower than the age of the patient in the current case and also lower than the median age of Japanese patients with ANCA-associated vasculitis (AAV) [33]. Additional involvement of the ears/nose/throat and lungs was reported in 17 and 11 of the 27 patients, respectively. In the present case, the ears, upper respiratory tract, and lungs were not involved. Anti-MPO and anti-PR3 antibodies were positive in 7 and 13 of the 20 ANCA-positive patients, respectively, whereas only two patients were double-negative for ANCA, similar to the findings recorded in the present case. Renal masses in the right, left, and both kidneys were present in 9, 11, and 7 patients, respectively, whereas solitary, multiple bilateral, and multiple unilateral renal masses were found in 17, 7, and 3 patients, respectively. Three patients progressed to end-stage renal disease. Renal histopathological examination revealed glomerulonephritis, granuloma, and vasculitis in 16, 23, and 10 patients, respectively. Regarding therapeutic approaches, partial and total nephrectomy was performed in 5 and 9 patients, respectively, and nephrectomy before diagnosis was performed in 14 of the 27 patients. Three patients underwent dialysis, and twenty-three patients were treated with prednisolone, cyclophosphamide, methotrexate, azathioprine, or rituximab. In addition, prednisolone and immunosuppressive treatment were effective for the management of all 23 patients with respiratory tract or renal lesions. In Japan, cyclophosphamide was used as induction therapy for AAV in only 39.6% of cases, which was lower than that reported in European countries [34]. Furthermore, the average age of Japanese patients with AAV was relatively high, and the most frequent cause of death was infectious complications [33]. Therefore, in Japan, reducing the dose of prednisolone for the initial treatment of patients with AAV, with or without the use of cyclophosphamide, is recommended [33]. Combination therapy (glucocorticoids and immunosuppressive agents) is currently recommended as the standard of care for patients with AAV in Japan. However, glucocorticoid monotherapy is being considered in high-risk patients, such as the elderly, people with renal failure requiring dialysis, and people highly predisposed to infection [35]. Boubenider et al. reported that renal function dramatically improved via treatment with prednisolone and immunosuppressive agents in patients with GPA and acute kidney injury without extrarenal manifestations [15]. The present patient was treated with low-dose prednisolone (30 mg/day) and did not receive cyclophosphamide because she was old. Nonetheless, she exhibited a good response to steroid therapy and could discontinue hemodialysis. Overall, the present case illustrates that GPA should be included in the list of differential diagnoses of solitary renal masses, regardless of ANCA status.

In conclusion, despite its rarity, GPA should be considered as one of the differential diagnoses of solitary renal mass in patients without prior respiratory manifestations, irrespective of their ANCA status. Severe narrowing due to neointimal thickening of the artery may worsen the injury of vasculitis in the localized area through endothelial ischemia, resulting in a renal mass. Patients with a solitary renal mass associated with GPA may show a favorable response to steroid or immunosuppressive treatment, even after nephrectomy.

## Data Availability

Not applicable.

## Abbreviations

GPA: Granulomatosis with polyangiitis
CHCC: Chapel Hill Consensus Conference
CRP: C-reactive protein
ANCA: Anti-neutrophil cytoplasmic antibodies
MPO: Myeloperoxidase
PR3: Anti-proteinase 3
AAV: ANCA-associated vasculitis
